# The Prevalence of Restless Legs Syndrome in Patients Undergoing Hemodialysis: A Systematic Review and Meta-analysis Study

**DOI:** 10.18869/nirp.bcn.8.2.105

**Published:** 2017

**Authors:** Reza Ghanei Gheshlagh, Mohammad Farajzadeh, Mozhdeh Zarei, Vajiheh Baghi, Sahar Dalvand, Kourosh Sayehmiri

**Affiliations:** 1- Social Determinants of Health Research Center, Kurdistan University of Medical Sciences, Sanandaj, Iran.; 2- Department of Nursing, School of Nursing & Midwifery, Kurdistan University of Medical Sciences, Sanandaj, Iran.; 3- Deputy of Research and Technology, Kurdistan University of Medical Sciences, Sanandaj, Iran.; 4- Department of Midwifery, School of Nursing & Midwifery, Kurdistan University of Medical Sciences, Sanandaj, Iran.; 5- Department of Biostatistics, Psychosocial Injuries Prevention Research Center, Ilam University of Medical Sciences, Ilam, Iran.

**Keywords:** Restless legs syndrome, Hemodialysis, Meta-analysis

## Abstract

**Introduction:**

Restless legs syndrome is a sensory-motor disorder that causes sleep disorder. The syndrome in patients undergoing hemodialysis associates with depression, sleep deprivation, performance disorder, day fatigue, excessive daytime sleepiness, stress, anxiety, and higher risk of cardiovascular diseases. The objective of this systematic meta-analysis study was to estimate prevalence of restless legs syndrome in patients undergoing hemodialysis.

**Methods:**

Twenty-six relevant articles published between 2000 and 2015 indexed in Iranian (MagIran and IranMedex) and international databases (SID, Google Scholar, ScienceDirect, PubMed, Pre Quest, and Scopus) were selected. Data analysis was carried out through meta-analysis (random effect model) and heterogeneity of the studies was determined using I2 index. The obtained data were analyzed in STAT (11.2).

**Results:**

Prevalence of the syndrome according to the found articles was 50% (95% CI: 38–61) in Iranian and 30% (95% CI:23–37) in international databases. There was an ascending trend of prevalence of the syndrome corresponding to the publication year of the articles (P=0.419), while the trend based on age of the patients was descending (P=0.604). However, the variations were not significant.

**Conclusion:**

Given the high prevalence and considerable effects of restless legs syndrome on patients undergoing hemodialysis, it is recommended that these patients be screened for the syndrome.

## 1. Introduction

Restless legs syndrome (RLS) or Willis-Ekbom is a chronic motor-sensory disorder characterized with intense desire to shake the legs during sleep (Al-Jahdali, 2011). The desire to shake the legs causes unpleasant feeling, which is described as high heartbeat, irritation or tingling, or as if insects are crawling on the legs. The symptoms are intensified when the patients are not physically active (Al-Jahdali et al., 2010; Bidaki et al., 2014). The international restless legs syndrome study group (IRLSSG) introduced 4 criteria to diagnose the syndrome; unpleasant sense of restless legs, desire to keep shaking the legs, aggravation of the symptoms at night and in the evening, and alleviation of the symptoms by shaking the legs (Anssarin, Argani, & Shaabanpour, 2008).

RLS is one of the most prevalent sleep disorders, which is not diagnosed in most the cases or the symptoms may lead to wrong diagnosis (Araujo et al., 2010). As suggested by the reports, out of 80% of RLS patients who seek medical attention, only 25% of the cases are properly diagnosed, and in turn only 13% receive effective medications (Atkinson et al., 2004). The syndrome is observed as idiopathic or secondary problem of other diseases such as rheumatology, diabetes, MS, neurological diseases, and renal problems (patients under dialysis in particular) (Bhowmik et al., 2003). The main causes of RLS are unknown; however, low serum iron and ferritin, changes in hormonal level of calcium, phosphate, and parathyroid and its consequences on nervous system are claimed responsible in development of the symptoms (Celle et al., 2009).

Prevalence of RLS is 2% to 20% (Bidaki et al., 2014; Cirillo, & Wallace, 2012) and studies have shown that it is considerably higher among patients undergoing hemo-dialysis (up to 60%) (Cuellar, Strumpf, & Ratcliffe, 2007; DeSimone, & Petrov, 2009). In addition to the unpleasant sense of restlessness of the legs, the syndrome is the root of other disorders such as depression, sleep deprivation, fatigue during the day, driving problems, problems in doing daily tasks, excessive daytime sleepiness, stress, and anxiety (Atkinson et al., 2004; Emami Naini, Masoumi, Mortazavi, Gholamrezaei, & Amra, 2012; Gade et al., 2013; Goffredo Filho, Gorini, Purysko, Silva, & Elias, 2003).

Moreover, the syndrome may cause problems in the dialysis process (Cirillo, & Wallace, 2012). Development of RLS among patients in need of dialysis tackles quality of life and the patient’s adaptation to dialysis; and along with psychological disorders, it causes serious issues such as cardiovascular diseases and increase of risk of death (Habibzade, Khalkhali, & Ghaneii, 2011). Knowing that programming and taking steps to prevent and treat the RLS needs accurate statistics about prevalence of RLS among patients under hemodialysis, the present study aimed to estimate the prevalence of the disease among patients undergoing hemodialysis.

## 2. Methods

This systematic review and meta-analysis study was carried out to measure prevalence of RLS syndrome among patients undergoing hemodialysis by reviewing articles indexed in international and Iranian journals. To this end, articles on the prevalence of RLS were searched in Iranian and international databases such as SID, MagIran, Google Scholar, IranMedex, ScienceDirect, PubMed, PreQuest, and Scopus. The keywords used in the search were “restless legs syndrome,” “symptoms of restless legs syndrome,” “hemodialysis,” combinations of these terms, and Farsi equivalents of the terms in case of articles published in Farsi. To extend the scope of the search, references of the articles found in primary search were also reviewed.

### 2.1. Inclusion criteria and data extraction

At first, all articles about the prevalence of RLS among patients undergoing hemodialysis were collected and irrelevant articles were removed based on exclusion criteria, including irrelevancy, medical interventions on the subjects, duplicate articles, and unavailability of the full text. In addition, studies on a combination of the hemodialysis and peritoneal dialysis or only peritoneal patients were excluded. Taking into account the inclusion and exclusion criteria, summary of the articles were examined by two independent researchers (RGG and MF) and a third researcher (expert in meta-analysis) settled any cases of disagreements. Totally, 26 articles published between 2001 and 2015 were selected based on inclusion and exclusion criteria.

### 2.2. Statistical analysis

Given that prevalence is a binomial distribution, variance of prevalence was calculated through binomial distribution variance. To combine prevalence data from different articles, weighted average was obtained so that weight of each article was inverse of the variance. To survey heterogeneity of the data, I2 index was utilized. Heterogeneity was categorized in 3 groups of low heterogeneity (25%), average heterogeneity (25%–75%), and high heterogeneity (>75%). Taking into account heterogeneity of the data (94.6%), random effects model was used. The relationship among prevalence of the syndrome, the number of subjects and year of publication was examined by meta-regression. Data analyses were carried out in STATA (11.2).

## 3. Results

**Figure 1.**
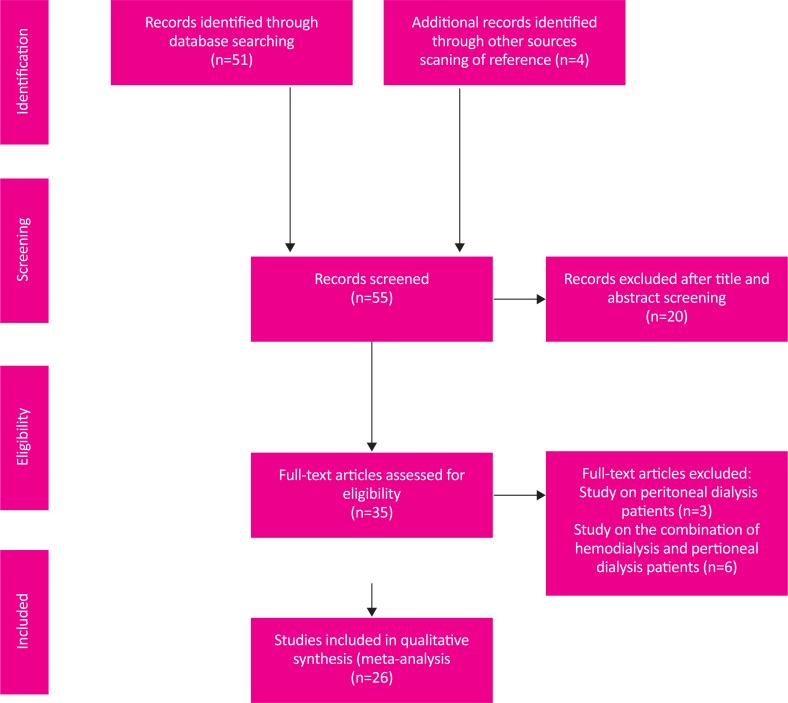
The process of surveying, screening, and selecting the articles for systematic review and meta-analysis.

All English articles published between 2000 and 2015 on RLS among hemodialysis entered the systematic review and meta-analysis process based on PRISMA instruction (Habibzadeh, Hemmati Maslakpak, & Ghanei Gheshlagh, 2012). Totally, 55 articles were selected through primary search and based on inclusion and exclusion criteria, 29 articles entered final analysis stage. The selection flow chart is pictured in Figure 1.

The articles entered the final review stage were those published from 2001 to 2014 and represented the total number of 6188 subjects (238 participants per study). The smallest sample group was in Shahid et al. (2010) (Habibzadeh, Lazari, & Ghanei Gheshlagh, 2013) study with 41 participants and the largest sample group was in Merlino et al. (Haider, Anees, & Shahid, 1969) study, with 883 participants. Highest and lowest prevalence rates of RLS were (68%) reported by Cotner et al. (Hemati, Alidosti, & Reisi, 2012) in the USA and (7%) by Biomik et al. in India (Higuchi et al., 2015). Fifteen articles (57.6%) were done in Asia; for further details of the articles see Table 1.

**Table 1.** Specifications of the articles in systematic review and meta-analysis of prevalence of RLS among patients undergoing hemodialysis.

**Authors**	**Year**	**N**	**Prevalence**	**95% CI**		**Country**	**Male (%)**
				**Lower Limit**	**Upper Limit**		
Higuchi et al.	2015	159	22	16	22	Japan	71
Wali et al.	2015	355	19	15	24	Saudi Arabia	91
Bidaki et al.	2014	160	67	60	74	Iran	51.1
Haider et al.	2014	250	65	59	71	Pakistan	61.2
Sharifi et al.	2013	80	57	47	68	Iran	51.2
Stefanidis et al.	2013	579	27	23	30	Greece	59.2
Hemati et al.	2012	171	57	50	65	Iran	55.6
Habibzadeh et al.	2012	168	39	31	46	Iran	48.2
Salman et al.	2011	123	20	13	27	Syria	56.9
Araujo et al.	2010	400	22	17	26	Brazil	59
Soyoral et al.	2010	76	14	7	22	Turkey	55.2
La Manna et al.	2010	100	31	22	40	Italy	63
Shahidi et al.	2010	41	17	6	29	Iran	51.2
Al-Jahdali et al.	2009	227	50	44	57	Saudi Arabia	53.7
Salimpour et al.	2009	130	33	25	41	Iran	63.1
Kim et al.	2008	164	28	21	35	Korea	56
Merlino et al.	2006	883	27	24	30	Italy	-
Mucsi et al.	2005	333	14	10	18	Hungary	58
Molahosseini et al.	2005	514	62	57	66	Iran	73.4
Mucsi et al.	2004	78	32	22	42	Hungary	-
Bhowmik et al.	2003	121	7	2	11	India	84.2
Takaki et al.	2003	426	16	13	20	Japan	60
Goffredo Filho et al.	2003	176	15	10	20	Brazil	60.8
Kutner et al.	2002	183	48	41	55	USA	51.9
Kutner et al.	2002	125	68	60	76	USA	45.6
Miranda et al.	2001	166	26	19	33	Chile	-

**Figure 2.**
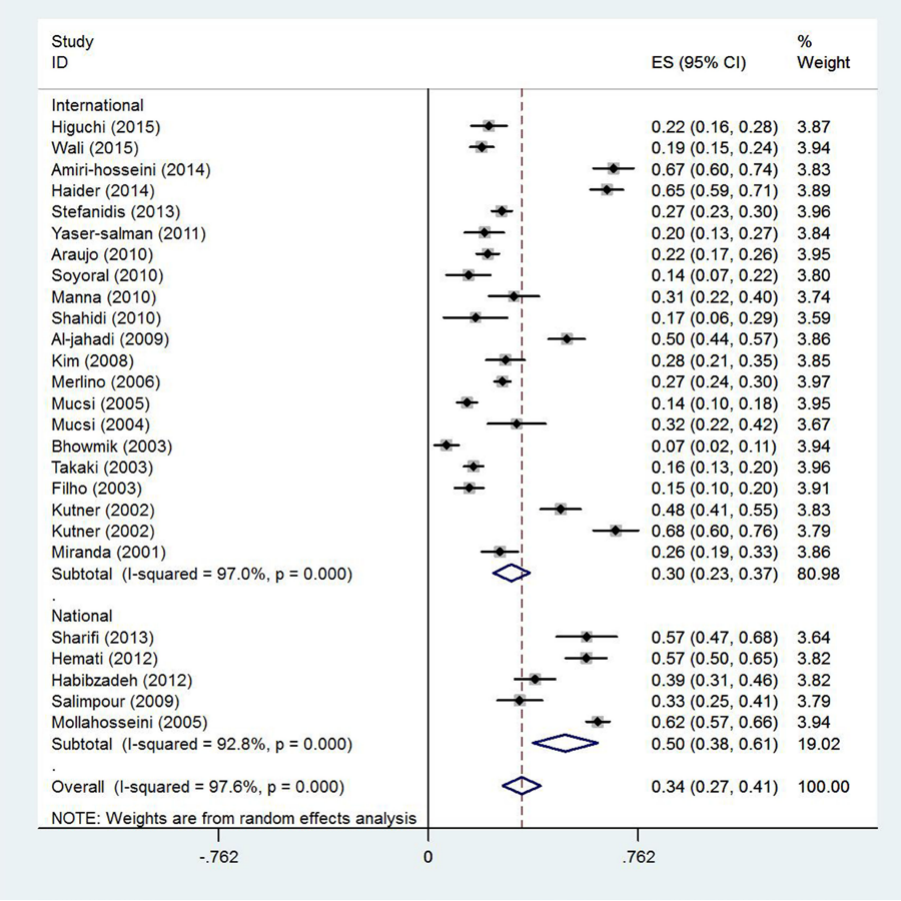
Prevalence of RLS based on the database. CI of 95% for each study is represented by horizontal lines near the main mean; dashed line at the middle of the chart indicates the total mean point; and the rhomboid represents CI of prevalence of the disorder.

**Figure 3.**
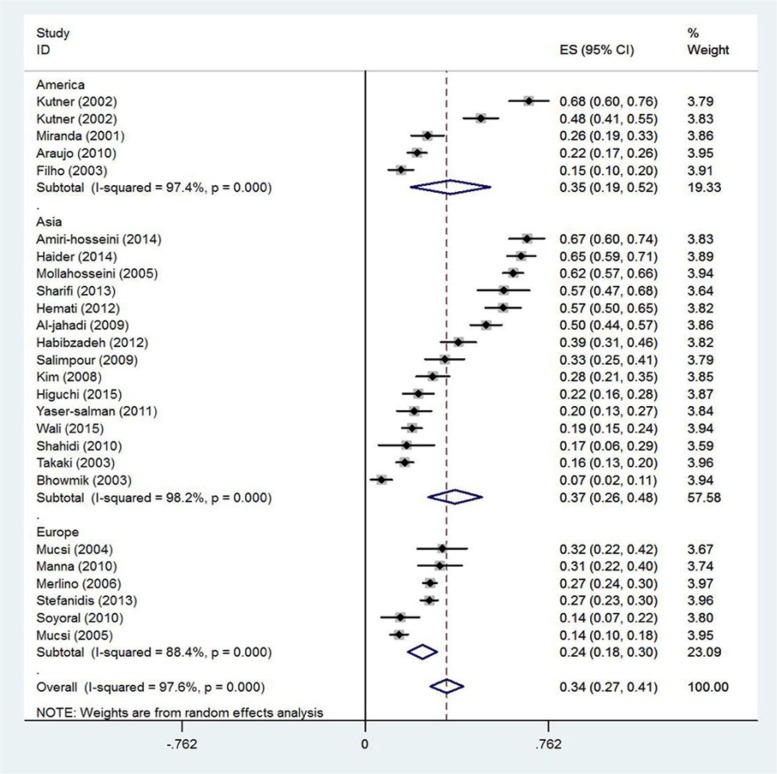
Prevalence of RLS based on place of study. CI of 95% for each study is represented by horizontal lines near the main mean; dashed line at the middle of the chart indicates the total mean point; and the rhomboid represents CI of prevalence of the disorder.

Given the high level of heterogeneity (97.6%) among the articles under study, random effects model was used. The model assumes that the differences between the results are due to differences in sampling process and the obtained scores by the subjects. Estimate of RLS prevalence among patients undergoing hemodialysis based on the random effects model was 34% for 6188 participants (95% CI: 27–41). The articles were categorized based on which continent the study was conducted (Figures 2 and 3).

**Figure 4.**
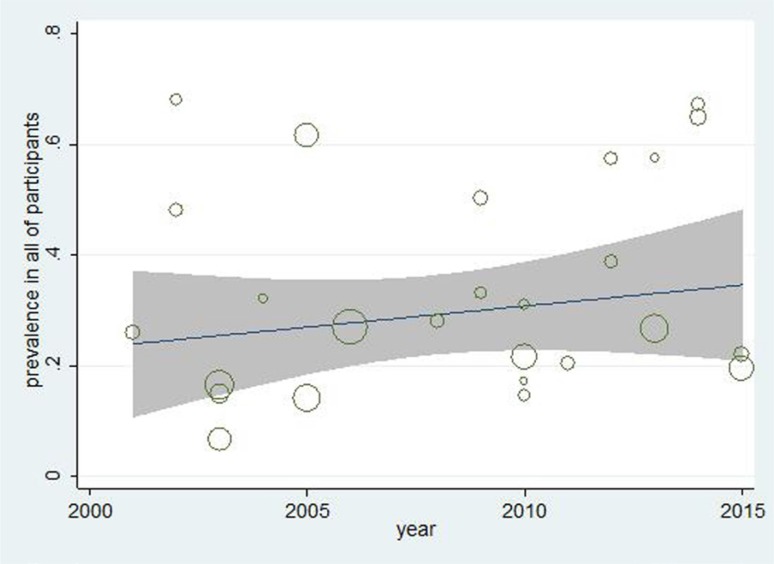
Meta-regression diagram of the prevalence of RLS based on the year of publication. The increasing trend is not significant. Each circle represents one article and the larger the circle the larger the study group.

**Figure 5.**
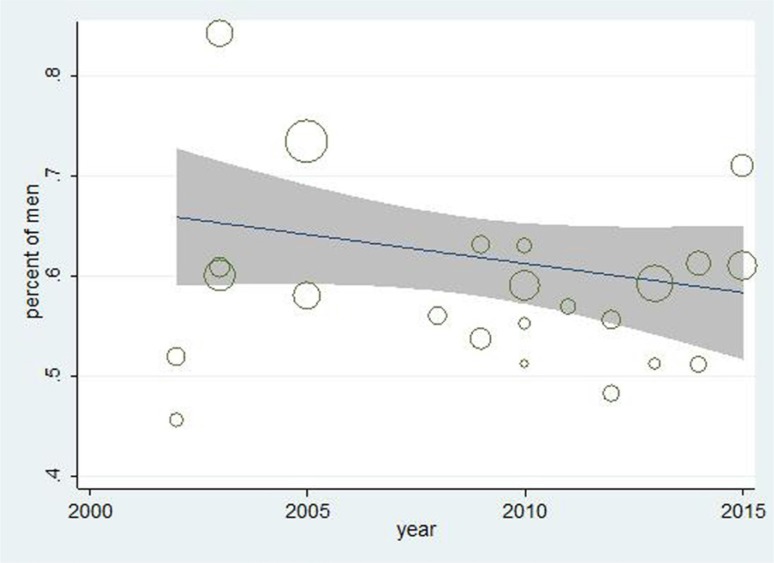
Meta-regression diagram of the prevalence of RLS based on the age of the patients undergoing hemodialysis. The descending trend is not significant. Each circle represents one article; the larger the circle, the larger the study group.

Meta-regression diagrams (Figures 4 and 5) indicate no relationship between prevalence of RLS in patients undergoing hemodialysis and year of article publication (P=0.419), and between prevalence of RLS in these patients and their ages (0.604). That is, the prevalence of RLS among patients undergoing hemodialysis does not change significantly based on their ages or year of article publication.

## 4. Discussion

Results of different studies on the prevalence of RLS in patients undergoing hemodialysis were examined and general prevalence of the syndrome among them was obtained as 34% (95% CI: 27–41). Prevalence of RLS in the patients undergoing hemodialysis was higher than that of healthy population; however, this is inconsistent with studies on hemodialysis and peritoneal dialysis patients. Emami Naini et al. (2012) reported the prevalence of RLS in patients undergoing hemodialysis as 35.5% and patients under peritoneal dialysis as 17.7% (Phillips, Hening, Britz, & Mannino, 2006). Al-Jahdali maintained that the prevalence of RLS among patients under hemodialysis (69%) was higher than that of patients under peritoneal dialysis (46%) (Samilipour et al., 2009). Decrease of renal function increases risk of RLS and only after kidney transplant, the risk of RLS returns to average level of normal population (Shahidi Namjo, & Najafzade, 2010). Studies have also shown that in patients with renal failure, symptoms of RLS syndrome may improve after kidney transplant and no considerable improvement is expected after dialysis (Sharifi et al., 2013). Although, the pathophysiological relationship between development of RLS syndrome and renal failure is vague, it appears that lack of vitamins, iron deficiency, and anemia are the key factors to blame.

RLS syndrome in hemodialysis patients leads to low life quality, high immunology and cardiovascular diseases, high blood pressure, sleep deprivation, daytime sleepiness, fatigue, daytime functions disorders. Anera notes that mortality risk of RLS hemodialysis patients is 1.4 times higher than that of the patients without RLS. In general, the hardships of hemodialysis process and the consequences in the form of decrease of performance, social seclusion, lack of physical activities, and decrease of self-confidence on one hand and the problems rooted in RLS syndrome on the other hand not only tackle quality of life of the patients but also problematize routine life order of the family members.

Prevalence of RLS in patients undergoing hemodialysis reported by articles found in the Iranian databases (50%; CI: 38–61) was higher than that by articles found in the international databases (30%; CI: 23–37). The difference between the reported figures might be due to racial, cultural, socioeconomic status, and medical services differences.

The results also showed that studies in Asia and America reported higher RLS prevalence compared to European studies. Although, the syndrome can be developed in all races, many authors believe that it is more prevalent in white races (Spolador, Allis, & Pondé, 2006). By referring to epidemiological studies, Atkinson (2004) stated that prevalence of RLS among European population was 5%–15% and this range for the North American population was 7.2%–11% (Stefanidis et al., 2013). Prevalence of the syndrome among African Americans and white Americans with renal failure was 48% and 68%, respectively (Sharifi et al., 2013). Racial differences, probability of developing the disease, environmental factors (geographical position), and methodological limitations (e.g. small sample group, contradictory interpretation of the measures) all may lead to different results regarding the prevalence of RLS.

Based on the meta-regression results, prevalence of RLS does not correlate significantly with age of the subjects. Inconsistently however, studies have shown that prevalence of the syndrome increases up to age 79 and afterward it follows a descending trend (Soyoral et al., 2010). Given age distribution of the subjects, average age of the subjects in each study is not a reliable index to measure relationship between the patients and prevalence of RLS. In this regard, individual patients’ data is a more reliable measure. Mucsi (2005) showed that age distribution of the patients with RLS and patients without RLS was not different (Moher, 2009). Regarding the relationship between the effects of age and race, Lee (2008) mentioned that high prevalence of RLS among European senior citizen population compared to African races, highlights the role of racial differences in this regard (Takaki et al., 2003). Unavailability of full-text of some of the articles was one of the main limitations of the present study.

RLS causes sleep disorder, sleepiness, and sleep deprivation in patients undergoing hemodialysis and leads to cardiovascular and immunological problems. Because, RLS symptoms might not be recognized as a clinical problem, it usually remains undiagnosed in hemodialysis patients.

In general, it can be concluded that prevalence of RLS is higher among patients undergoing hemodialysis compared to general population and prevalence of the syndrome has not changed over the recent years. By diagnosing the hemodialysis patients with RLS, the unpleasant symptoms and problems caused by the symptoms can be alleviated.
